# Early Intervention of Hemiplegic Shoulder Pain in the Context of Post-Stroke Shoulder Spasticity: A Canadian Cross-Sectional Survey

**DOI:** 10.3390/toxins18050228

**Published:** 2026-05-12

**Authors:** Farris Kassam, Fraser MacRae, Linden Lechner, Heather Dow, Ève Boissonnault, Fiona Huang, Paul Winston

**Affiliations:** 1 Faculty of Medicine, University of British Columbia, Vancouver, BC V6T1Z3, Canada; 2 Canadian Advances in Neuro-Orthopedics for Spasticity Consortium, Kingston, ON K7K1Z6, Canada; fmacrae2@uwo.ca (F.M.);; 3 School of Physical Therapy, Western University, London, ON N6G1H1, Canada; 4 Faculty of Medicine, University of Alberta, Edmonton, AB T6G2R7, Canada; 5 Department of Rehabilitation Medicine, Vancouver Island Health Authority, Victoria, BC V8R1J8, Canada

**Keywords:** spasticity, hemiplegic shoulder pain, botulinum toxins

## Abstract

Objectives: To investigate current Canadian physicians’ practice patterns of treating upper limb post-stroke spasticity (PSS) and hemiplegic shoulder pain (HSP) acutely after a stroke. In addition, by examining Canadian physicians’ diagnostic capabilities, time till treatment, minimum criteria to begin treatment, mechanisms of treatment, targeting of muscles, and benefits and adverse effects of treatment, we aim to learn about areas of improvement to optimize PSS management for Canadians. Design: The present study was a cross-sectional survey, polling practicing Canadian physicians. Results: A total of 17 physicians completed the survey, all PM&R specialists, save one neurologist. Four provinces were represented in the responses. Participants had, on average, over ten years of experience managing post-stroke spasticity in outpatient and inpatient clinics. All 17 perform botulinum neurotoxin A (BoNT-A) injections for HSP associated with PSS. Most participants reported that they will begin BoNT-A treatments 2–3 weeks post-stroke, most commonly targeting the pectoralis major, subscapularis, and latissimus dorsi. Participants reported the mean median dosage they use as onabotulinum toxin A (169.12 units, SD = 73.70), incobotulinum toxin A (178.13 units, SD = 65.75), and abobotulinum toxin A (470.83 units, SD = 171.17). For injection guidance, participants responded that they use ultrasound for the largest percentage of their caseload, followed by electromyography, then electrical stimulation, then palpation. Very seldom did participants use palpation alone. Conclusions: From the limited sample included in analyses, the Canadian physicians respondents seem to be treating HSP and associated PSS with variable strategies. Further research is required to align dosages, targets, and guidance strategies as they vary considerably.

## 1. Introduction

Stroke is the second largest cause of death, with an annual global mortality rate of 6.5 million people [1]. The advancement of therapies means that a majority (up to 85%) of stroke victims survive, but over 50% of these patients are left chronically disabled, relying on their friends, families and health care system for support [2]. Hemiplegic shoulder pain (HSP) is widely recognized as a common complication contributing to disability after stroke, with a recent study showing 56% of 480 strokes in a rehabilitation unit suffered from shoulder pain [3]. Spasticity is a common sequelae following stroke that presents in up to 43% of patients [4]. It is defined as “involuntary muscle hyperactivity in the presence of central paresis. The involuntary muscle hyperactivity can consist of spasticity sensu strictu, of rigidity, of dystonia and of spasms or a mixture of those elements” [5]. Focal chemodenervation with Botulinum toxin type A (BoNT-A) is increasingly utilized in clinical practice to address muscle overactivity in post-stroke spasticity (PSS), yet the role in the management of early post-stroke HSP has not been clearly defined.

Management of PSS can be particularly challenging, as the onset of PSS can appear acutely following a stroke or present later in the management of chronic stroke [4]. Since effective treatment is based on early recognition and intervention, physicians must constantly be aware of its presentation in order to reduce progression and improve patient outcomes [6]. A delay in the identification and treatment of PSS can result in muscle shortening and tendon and soft tissue contractures [7]. Contractures are an important effect as they can cause pain, deformities of a patient’s limbs, and extreme limitations in a patient’s activities of daily living [8]. Once contractures present, spasticity can be very difficult to treat [7].

Like PSS, the onset of HSP can be acute, with 17% of patients experiencing HSP within 1 week. As in PSS, it is suggested that early intervention can reduce the risk of contracture and maximize recovery [8,9,10]. The pathology of HSP can be multifaceted; it can include musculoskeletal pain generators such as acromioosteoarthritis, adhesive capsulitis, rotator cuff of biceps tendinopathy, subacromial bursitis, shoulder impingement syndrome, glenohumeral subluxation, complex regional pain syndrome (CRPS), and neurological contributors including central neuropathic pain, and one of the main contributors of HSP, spasticity [11,12]. It is important to consider many possible simultaneous processes.

The 2023 national survey by Kassam et al. described Canadian practices related to BoNT-A use for shoulder spasticity across mixed etiologies, primarily in chronic PSS populations, with the largest group being Physical Medicine and Rehabilitation [13,14]. However, the study did not examine the etiology of early HSP, time-to-intervention after stroke, patterns of adjunctive and non–BoNT-A treatments, or the unique muscle-targeting considerations of the acute and subacute phases. To address these gaps, we developed and deployed a focused survey among Canadian physicians engaged in early-phase post-stroke care. It is designed with a primary outcome to capture not only BoNT-A injection practices, but also (1) diagnostic strategies and clinician confidence in distinguishing spasticity-driven HSP from other etiologies in the early post-stroke interval; (2) decision thresholds and minimal time-to-treatment post-stroke; (3) muscle selection specific to the early post-stroke shoulder, which may differ from chronic spastic patterns; (4) use of non-BoNT-A interventions (e.g., glenohumeral or subacromial injections, nerve blocks, cryoneurolysis, other pain procedures); (5) access to and use of adjunct physiotherapy or rehabilitative modalities; and (6) patterns of follow-up, outcome measurement, and goal-setting practices. By concentrating specifically on the early post-stroke window, this study provides characterization of real-world clinical practices for emerging shoulder spasticity and HSP.

## 2. Results

### 2.1. Participant Demographics

**Figure 1:**
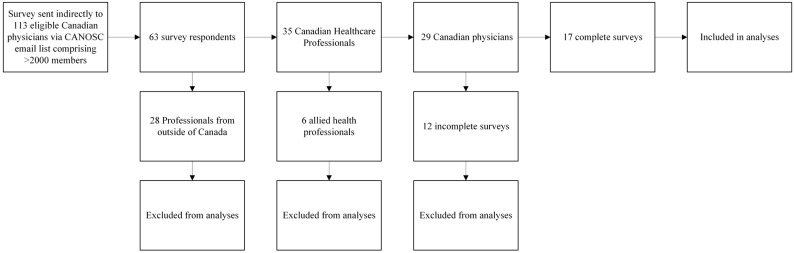
Summary of the inclusion process. Participants who practiced outside of Canada or were not physicians are excluded from analyses. Participants are also excluded if they failed to provide any answers to questions outside of demographic information.

The participants in this study were licensed Canadian physicians who treat PSS and HSP. In all, 17 participants completed the survey. Prospective participants were excluded if they neglected to continue the survey beyond providing demographic information, were practicing in countries other than Canada, or were allied health professionals other than physicians. Figure 1 contains a flowchart of the recruitment strategy, response rates, and exclusion criteria. Given that CANOSC comprises 113 Canadian physicians, the complete response rate was 15%.

Of the 17 participants, all were physical medicine and rehabilitation specialists, save one neurologist. Four provinces were represented by the survey respondents: seven reported that they are practicing in Ontario, four in British Columbia, three in Alberta, and three in Quebec. The sample had a mean of 10.59 (SD = 6.18) years of treating PSS and a mean of 10.18 (SD = 6.06) years of treating HSP. They reported that they had been treating HSP with BoNT-A injections for a mean of 10.69 (6.12) years and had been treating PSS with BoNT-A for a mean of 10.41 (6.05) years. All 17 participants reported working in an outpatient clinic, 15 of them also worked in an inpatient rehabilitation unit, 6 in an acute care setting, and 6 in long-term care. Two respondents reported that they work with children and adults; the remainder work only with adults.

### 2.2. Distinguishing HSP

Participants were asked to rank the most common causes of HSP in their practices. Overall, the highest-ranked answer was the stroke itself (weighted score = 172, mean score = 1.7, *n* = 17), followed very closely by spasticity (weighted score = 170, mean score = 3.4, *n* = 17). Next most important were adhesive capsulitis (weighted score = 152, mean score = 3.9, *n* = 17) and rotator cuff pathology (weighted score = 144, mean score = 2.9, *n* = 17). When asked about the ability to accurately differentiate the cause of HSP being PSS as opposed to other etiologies such as adhesive capsulitis, other glenohumeral pathology, rotator cuff pathology, myofascial pain, contracture, heterotopic ossification and CRPS, only four of seventeen respondents stated that they could confidently isolate the etiology with 80–100% certainty. The respondents indicated that the clinical signs and symptoms that would most shift their pre-test probability towards HSP being due to spasticity over other causes were flexor patterning in the upper limb (13 participants), limitation of shoulder abduction (11 participants), limitation of shoulder external rotation (10 participants), pain on passive movement (9 participants), pain on active movement (5 participants), limb malalignment (3 participants), pain at rest (2 participants), night pain (1 participant), pain on palpation (1 participant), pain as a dull ache (1 participant).

Five participants indicated that they would utilize a diagnostic nerve block to differentiate spasticity from contracture. All five reported that they will block the lateral pectoral nerve to pectoralis major; four also block the medial pectoral nerve to pectoralis minor; two also block the thoracodorsal nerve to latissimus dorsi, and three also block the subscapular nerve to teres major or subscapularis. Of the twelve respondents who do not perform diagnostic nerve blocks, eleven reported that lack of clinician training was a contributing reason.

### 2.3. Clinical Treatment of HSP

**Figure 2:**
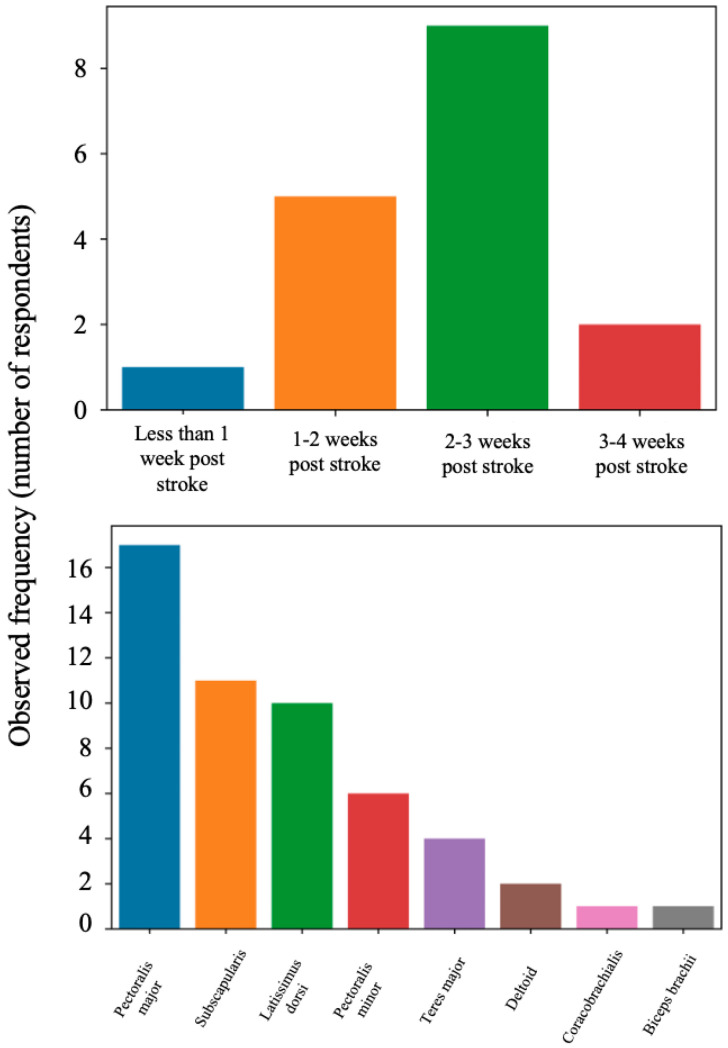
Summary of intervention timing and targets by observed frequency. The bar plots show the frequency that clinicians reported they would start treatment with BoNT-A injections (**top**) and the targets that they are most often choosing for BoNT-A treatment (**bottom**).

All 17 of the study participants reported that they perform BoNT-A injections for HSP treatment associated with shoulder spasticity. They reported that they use BoNT-A as a first-line treatment for spastic upper extremities for a mean 64.18% (SD = 28.57%) of their patients. Participants reported the minimum MAS rating at which they would consider administering BoNT-A injections in the shoulder at a median of 2 (IQR = [1,2], *n* = 17). They would consider beginning treatment less than a week post-stroke (one participant), 7–14 days after stroke (five participants), 15–21 days after stroke (nine participants), 22 days–1 month after stroke (two participants). A summary of the timing of BoNT-A interventions is included in Figure 2. The most treated muscles for PSS were pectoralis major (17 participants), subscapularis (11 participants), and latissimus dorsi (10 participants). Also mentioned were pectoralis minor (six participants), teres major (four participants), deltoid (two participants), coracobrachialis (one participant), and biceps brachii (one participant). A summary of the muscles targeted is provided in Figure 2.

Participants reported some adverse effects after BoNT-A injections, including weakness (10 participants), pain, swelling, redness, or bruising at the treatment site (7 participants), flu-like symptoms (2 participants), and denervation or atrophic changes in the muscle over time (1 participant).

In terms of treatment dosage, participants were asked to estimate the minimum, median, and maximum dosages used for onabotulinum toxin A, incobotulinum toxin A, and abobotulinum toxin A separately (owing to differences in dilutions between strains). The means and standard deviations of each dosage strategy are reported in Table 1. These data cannot be compared as the units are not equivalent between strains. Further, this data is not intended as a treatment guide and is purely informative as it is derived from a small sample and acquired by participant reporting without explicit tracking practices. Notably, as the dosages increased, so did the variability between responses. Participants reported that the metric they primarily use for determining target muscles and dosages was clinician preference (6 participants), MAS (5 participants), patient goals (5 participants), and clinical reasoning and experience (1 participant).

**Figure 3:**
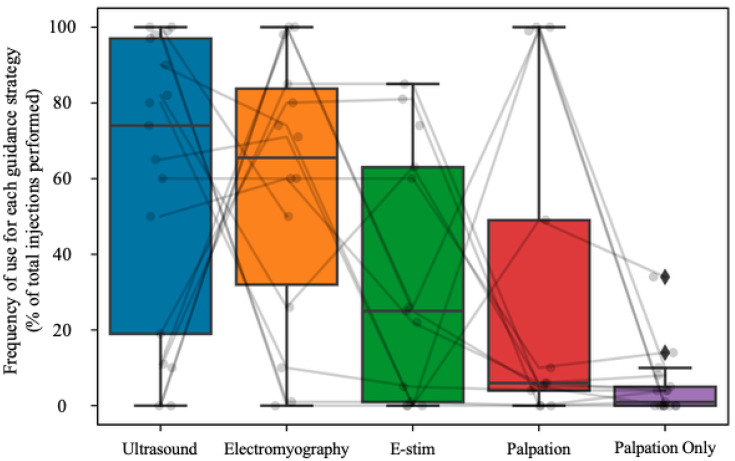
A box-and-whisker plot showing guidance technique preferences at a group level with individual responses overlaid. The frequency at which participants reported using each guidance strategy expressed as a percentage of their total injections is shown on the *Y*-axis. The boxes show the interquartile range (IQR), the horizontal line in the boxes shows the median, and the whiskers show the variability of the data outside of the IQR. Outliers are shown as black diamonds. Individual responses are shown as translucent black points, connected within each participant as translucent black lines. The points are jittered for clarity. All 17 participants respond to indicate how often they use ultrasound guidance, and how often they use palpation only. Just 14 participants report how often they use electromyography, and 13 report how often they use electrical stimulation or palpation.

While performing BoNT-A injections, participants reported that they use ultrasound guidance for the highest proportion of their injections at 60.88% (SD = 38.25%, *n* = 17). They estimated using electromyography for a mean of 58.21% (SD = 35.93%, *n* = 14) of their injections. They also reported using electric stimulation a mean of 34% (SD = 33.68%, *n* = 13) and manual palpation 29.54% (SD = 41.93%, *n* = 13) of the time. Finally, they reported using manual palpation only just 4.94% (SD = 8.57%, *n* = 17) of the time, with eight of those clarifying that they never perform injections with manual palpation only. Correlational analysis of the participants’ reported guidance technique preferences was conducted using data from all participants who reported the rate at which they use each guidance technique probed (zeros included) for a total of 12 participants considered. There was a strong, significant negative correlation in the rate at which clinicians used EMG guidance and US guidance (Pearson r = −0.84, *p* = 0.0006), indicating that participants who used US guidance frequently used EMG more rarely and vice versa; no other correlations were significant. This finding should be interpreted cautiously as it is derived from a small sample size and relies on participant-reported data alone. This finding highlights the hypothesis that clinicians who are more likely to use US are less likely to use EMG guidance. Further study is required to understand the nexus between guidance techniques in spasticity injections. A summary of the guidance technique preferences of the group, with individual data overlaid, is provided in Figure 3.

The group was polled on how often they use several alternative medical interventions for HSP. Participants reported that they offer subacromial or glenohumeral injections of corticosteroids and anesthetics for a mean 59.12% (SD = 22.40%, *n* = 17) of their patients. They report that they would offer suprascapular nerve blocks to a mean 22.88% (SD = 32.92%, *n* = 16) of their patients. Finally, the group reported that they would offer suprascapular nerve radiofrequency, neurotomy, or cryoneurolysis for 13.13% (SD = 24.60%, *n* = 16) of their patients, though notably, all participants reported at most 11% of their patients, save 3 clinicians who drove the effect.

### 2.4. Adjunctive Therapies

Participants stated that a mean 54.18% (SD = 23.32%, *n* = 17) of BoNT-A treatment and rehabilitation programs were applied concurrently at their centers, and they reported that a mean 41.59% (SD = 32.06%, *n* = 17) of their adult patients with spastic paresis can receive adjuvant, institutionally funded physiotherapy in combination with BoNT-A treatment. For adjuncts, all 17 participants reported that on average, the most used treatments were stretching (73.12%, SD = 34.01), active exercise programs (67.12%, SD = 26.38), and explicit referral to physiotherapy (65.41%, SD = 23.21). Of note, most of the common adjunct treatments fell within the scope of physiotherapy (taping, electrotherapy (FES, NMES), stretching, active energy program). Barriers to survey respondents seeking adjunct treatments included clinician-based time constraints (10 participants), risk of adverse events (4 participants), lack of evidence (3 participants), and patient preference (2 participants).

### 2.5. Follow-Up Evaluations Post-BoNT-A Treatment

All participants reported that they reviewed their patients with HSP in follow-up. They report that follow-up occurs at a mean of 2.77 weeks (SD = 2.98) after injections in an inpatient setting, and a mean of 7.88 weeks (SD = 3.66) in an outpatient setting. To track patient progress, respondents report using the MAS with 76% (SD = 31%) of their patients, tracking range of motion with 73% (SD = 33%), and assessing strength with 53% (SD = 41%). Other notable progress monitoring strategies included using a goal attainment scale (34%, SD = 33%), and a verbal rating scale (36%, SD = 47%). Participants report using clinical goal setting with 88.13% (SD = 15.77%) of their patients. On average, participants reported spending 10.69 min (SD = 8.57) on goal setting with their patients.

## 3. Discussion

As other cross-national surveys have provided insight into the management of upper limb spasticity, our study focused on HSP in the context of PSS with additional consideration to identifying what other pathologies are considered, identified and treated. This is a follow-up study to our original paper that examined Canadian Physicians’ Use of Intramuscular BoNT-A Injections for Shoulder Spasticity [13]. In our current study, we looked at HSP in the context of PSS and investigated Canadian BoNT-A treatment practices and adverse effects. We also looked at alternative pathologies of HSP and how they are treated with adjunctive therapies, outcomes of treatment, and future implications. Our participants were experienced practitioners in the treatment of PSS and HSP, with 10.59 (SD = 6.18) mean years of treating PSS and 10.18 (SD = 6.06) mean years treating HSP.

This survey reveals that physicians who responded that they are managing early HSP in the context of PSS employ a wide range of diagnostic and therapeutic strategies, with the recognition of the importance of early intervention. While all respondents use botulinum toxin type A (BoNT-A) to treat spasticity-related HSP, differences emerged in diagnostic confidence, in differentiating the causes of HSP, timing of treatment, muscle selection, and preferred guidance modalities. In addition to BoNT-A, clinicians reported a wide range of additional medical interventions targeting pain and mechanical contributors to early HSP. Subacromial or glenohumeral corticosteroid and anesthetic injections were the most frequently used, offered to a mean of 59.1% of patients, likely reflecting their role in managing inflammatory and capsular pathology that often coexists with spasticity. Suprascapular nerve blocks were used less commonly, with clinicians providing them to only 22.9% of patients on average. More advanced neuro-interventions—including suprascapular nerve radiofrequency ablation, neurotomy, or cryoneurolysis—were used in 13.1% of patients overall; however, this use was heavily skewed by three high-utilization clinicians, with most respondents using these procedures in fewer than 11% of cases. Taken together, these findings suggest that early HSP in PSS care among this group of physicians may be characterized by early use of BoNT-A but inconsistent diagnostic confirmation, variable access to adjunctive therapies, and diverging injection-guidance philosophies. It underscores that there is no unified approach to the management of HSP. This is not unique to Canada, as international studies also show variability in the treatment of PSS and HSP by practitioners [15,16]. This study had a majority of participants (83.1%) who had only performed three or fewer interventions in the last 3 months to treat HSP. The study (see above) noted that applying various means to relieve shoulder pain will be conducive to the recovery of upper limb motor function and shorten the in-hospital rehabilitation time. We found that clinicians generally chose either ultrasound or EMG as their main localization method, rather than using them together. The other localization strategies did not show any clear pattern of use. This may reflect differences in training, access to equipment, and clinic workflow. This was previously noted by MacRae et al. in 2024 [17].

Most clinicians reported concurrent BoNT-A and rehabilitation programs; however, only 41% of patients had access to institutionally funded physiotherapy paired with injections. Adjunctive practices (stretching, active exercise, taping, electrotherapy) were commonly used, and all fall within the physiotherapy domain—yet clinician time constraints were the single most frequently cited barrier to accessing these therapies. Clinicians reported spending an average of 10.69 min on formal goal setting with patients, reflecting the time constraints commonly encountered in clinical practice. Limited time for counseling may also contribute to the observation that 11.76% of clinicians attributed lack of adjunctive therapy use to patient preference, despite established evidence supporting the benefits of multimodal rehabilitation. These findings underscore that although optimal management of early HSP is multimodal, access to adjunctive therapy and comprehensive education about its value remain inconsistent across our cohort. Follow-up intervals differed markedly between inpatient (approximately 3 weeks) and outpatient (8 weeks) settings. MAS scores and ROM were the most used outcome measures, but the adoption of goal-oriented approaches was high, with nearly 90% of clinicians using structured goal setting.

Our results show that there is a persistent difficulty in differentiating spasticity-driven HSP from other causes of shoulder pain. Despite being an experienced cohort, only a small proportion of clinicians reported high diagnostic certainty. Diagnostic certainty for identifying spasticity as the primary cause of HSP was low, with only 4 clinicians reporting ≥80% confidence, and 5 respondents performing diagnostic nerve blocks—most commonly targeting the lateral pectoral nerve—while 11 of 12 non-users cited lack of training as the barrier. The low usage of diagnostic nerve blocks—performed by only five respondents, with the majority (64.71%) citing lack of training—suggests that a potentially valuable confirmatory tool remains a novelty in specialized clinics. This was also noted by another Canadian paper by Fitterer et al. in 2021 highlighting that progress still needs to be made to standardize treatment [18].

Flexor patterning, limited abduction/external rotation, and pain on passive movement were the most influential clinical features suggesting a spastic etiology. Adjunctive therapies and follow-up practices likewise varied, influenced by access and system limitations. Given that HSP is so common after stroke and one of the most common complications of stroke, these findings reinforce the need for clearer, evidence-based pathways for early management of HSP in the context of PSS across Canada.

Just as in Sheean 2010, while we note that HSP in the context of PSS requires an individualized treatment intervention, we noted that the intervention is widely varied with respect to muscles targeted, timing of intervention, doses used, timing of follow-up, the use of diagnostic nerve blocks and the recommendations of adjunctive therapies [19]. Similarly, Baguley 2011 found that muscle selection and doses were predominantly based on physician preference as opposed to objective measures such as degrees of spasticity, highlighting the need for increased exploration of physician practices so that more standardized treatment practices can be obtained for consistency of care in Canada [20].

Variability also exists with respect to the use of BoNT-A both nationally and internationally. BoNT-A interventions, having been shown to improve maladaptive positioning of limbs [21], posture and gait [22,23,24], reduce predetermined disability parameters, pain [25] and caregiver burden [26]. However, it is not universally identified as a first-line treatment for spastic upper extremities [20,21,22,23,24,26] although Canadian Stroke Best Practice Recommendations endorse botulinum toxin for the management of focal HSP (Heart and Stroke Foundation of Canada) [27,28].

While most of our participants (64.18%) believe that BoNT-A injection is the first-line treatment for spastic upper extremities, this was not unanimous. This is in keeping with other studies, such as Baricich 2021, who coordinated a panel of 21 Italian specialists to take part in a web-based survey Delphi process [29]. Similar to our findings, the expert panel found that a majority (83%) but not all of its members believed that BoNT-A is a first-line treatment as soon as post-stroke spasticity appears. In the UK, however, Holmes 2019 found that only 8.8% of physicians believe that BoNT-A was a first-line treatment, highlighting how a lack of clinical guidelines to treat HSP in the context of PSS can lead to variability internationally [15].

While pectoralis major was overwhelmingly the primary muscle target in the treatment of spasticity, which is congruent with similar findings in the literature, subsequent targeting of upper limb muscles was also varied [13,15]. When comparing to a similar Canadian study that also sampled from CANOSC in 2023, the order of frequency of injection was pectoralis major, latissimus dorsi, pectoralis minor and subscapularis which were treated in similar frequencies and finally, teres major [13]. Contrastingly, the results of our study revealed a frequency of pectoralis major, subscapularis, latissimus dorsi, pectoralis minor, teres major, deltoid, and finally, coracobrachialis and biceps brachii at similar rates. Here, subsequent muscles targeted, along with muscles not included in Kassam et al., emphasize the evolving nature of the field since Kassam et al. was also sampled from CANOSC in 2023 [13]. Similarly, Holmes and Connell’s participants injected an even longer list of muscles, 15 in total, further highlighting variability in treatment for HSP from healthcare practitioners across the globe.

Most respondents initiated BoNT-A treatment early after stroke, with nearly 90% reporting intervention within the first 2–3 weeks. The median MAS score prompting treatment was 2; more than one-third would consider treatment at lower MAS thresholds. This likely reflects clinical decision making that accounts not only for tone severity but also for pain, hygiene considerations, positioning needs, muscle shortening and the prevention of contracture.

The Canadian early-treatment practices are consistent with recommendations to treat by three months, and tend to be even earlier, as all our respondents believe that intervention should occur at no longer than 1 month. Italian experts, via a Delphi consensus process, unanimously agreed that PSS should be identified at the earliest opportunity, and patients should be closely monitored for ensuing tonicity changes and suggested mandatory monitoring for at least one year following stroke [29]. Since spastic hypertonia can emerge early after stroke, early detection and intervention can prevent the formation of contractures and deformities [30]. When examining when our participants provided intervention, an almost bell-shaped distribution presents (Figure 2) when examining our participant’s belief as to when to begin treatment after stroke: 5.88% of participants believe in intervention less than a week post-stroke, 29.41% of participants believe in intervention 7–14 days after stroke, 52.94% of participants believe in intervention 15–21 days after stroke, and 11.76% of participants believe in intervention 22 days–1 month post-stroke. Picelli et al. defined early intervention as up to 3 months post-stroke [31].

While there has been variation with respect to the treatment practices of where and when to inject BoNT-A for patients with early intervention of HSP in the context of PSS, since we have an experienced cohort, unsurprisingly, there is minimal variability regarding the adverse effects after BoNT-A injection. Knowing the desired effect of BoNT-A, it is unsurprising that a majority (58.82%) of participants noted weakness as being the main side effect of BoNT-A injection. As a testament to the quality of care from the respondents, a minority (41.18%) of participants noted benign side effects such as pain, swelling, redness or bruising. Unlike Holmes and Connell, no major side effects such as dysphagia occurred.

### Limitations

This study has several limitations. The sample size was small and limited to clinicians affiliated with CANOSC, which may overrepresent clinicians with experience and interest in early spasticity management. The lower responses compared to our first study, Kassam et al. in 2023, which had triple the respondents, suggest that the complexity of advanced management may have reduced the number of respondents [13]. There could also be a response bias in which participants’ answers may reflect their impressions of expected practice, rather than their own clinical activity. We attempted to attenuate this bias by the use of anonymity of responses and the omission of recorded clinician-specific identifiers. Inevitably with voluntary survey administration, self-selection bias must be addressed as clinicians with an interest in this topic are more likely to participate in this study leading to an underrepresentation of the targeted sample; thus, this bias must be considered when interpreting results [32]. The survey also relied on self-reported practices rather than direct observation or patient-level outcomes. Further, although precautions such as revision and review of survey questions were taken to ensure readability and clarity, participants may have misinterpreted the phrasing of questions leading to frustration and could explain why some subjects decided to leave the survey only after completing the demographics section. Additionally, we recorded no responses in the territories of Canada, as this is likely due to the lack of permanently practicing clinicians in these regions [33].

## 4. Conclusions

The findings of this study demonstrate that the clinicians who responded recognize the importance of early management of HSP in the context of PSS, but differ substantially in diagnostic approaches, timing, muscle targeting, and adjunctive care. Variability appears driven by differences in training, clinic resources, physiotherapy access, and reliance on personal clinical experience though the sample of physicians included seems to represent a very small expert group and may not be representative of national trends. Our findings show that the pathway to standardizing diagnosis, improving access to concurrent physiotherapy, and developing early-phase treatment algorithms remains unclear.

## 5. Methods

### 5.1. Study Design

A cross-sectional survey of Canadian physicians with experience in spasticity management and HSP was designed and then approved by the local institutional research ethics board.

### 5.2. Survey Design

The survey was developed with the input of the senior author (PW), an attending physician (EB), and two medical students (FK and LL). The survey contained a mixture of multiple choice, rank order, percentage and open-ended questions. The final version consisted of a total of 50 questions. The survey included both quantitative and qualitative responses.

### 5.3. CANOSC

The survey was sent to eligible Canadian physicians using the Canadian Advances in Neuro-Orthopedics for Spasticity Consortium (CANOSC) mailing list. Details about CANOSC and its membership can be found in a previous publication that also used this platform to recruit for their survey [13]. A 2019 survey spearheaded by the Canadian Medical Association estimated there were 500 Canadian PM&R specialists in clinical practice [14]. CANOSC has 113 Canadian Physiatrists, approximately 22.6% of all active PM&R physicians in Canada, and a majority of all Canadian PM&R physicians who treat PSS and HSP. These demographics make it an ideal organization to examine PSS and HSP treatment practices in Canada.

### 5.4. Participant Recruitment

The sample population for this study was Canadian physicians involved in the management of PSS and HSP. An invitation email was sent to CANOSC members using an online link. The email contained information about our study, obtaining consent, privacy information, ethics statements, and the link to the anonymized survey platform. To ensure the anonymity of responses, the online survey tool did not record any identifying information. There were no offers of incentives to participants.

### 5.5. Data Analysis

The survey was hosted on the web-based platform, Alchemer (www.alchemer.com accessed on 3 January 2026, Louisville, CO, USA). Primary data was securely stored on this web-based platform. The CAPMR (Canadian Association of Physical Medicine and Rehabilitation) secretariat has an enterprise license for this software.

Raw data was exported from Alchemer as a Microsoft Excel (v.16.103.1) spreadsheet. Correlational analyses and descriptive statistics were computed using Python (v.3.11.7) with packages Pandas (v.2.1.4) and Numpy (v.1.26.4) for data manipulation, matplotlib (v.3.8.0) and seaborn (v.0.11.0) for data visualization, and Scipy (v.1.11.4) for analyses. The analysis code and dataset are available upon reasonable request to the authors. Descriptive statistics are presented as means with standard deviations for parametric data and medians with interquartile ranges for non-parametric data. Where applicable, weighted scores were computed such that participants ranked all list items; items ranked as 1 received a score of *n* where *n* is the number of list items. Items ranked as second received a score of *n* − 1, and so on. Correlational analyses were conducted using Pearson’s r for parametric data. The number of responses for each question is reported throughout the text; the only time questions were combined was for a single correlational analysis. Participants who failed to answer any of the questions involved in the correlational analysis were excluded from it.

The questionnaire was thoroughly reviewed by all the study authors and a data analyst during development. Any disagreements were resolved through consensus. Canadian physicians who finished the survey were included in analyses for the questions they completed. This explains why questions may have different numbers of responses. The survey platform was open between March 2025 and September 2025.

## Figures and Tables

**Table 1:** Mean minimum, median, and maximum reported dosages for botulinum toxins by strain. Toxin units across strains are not comparable as units are not equivalent and titrations differ between strains.

	Group Mean Onabotulinum Toxin A Dosage (SD)	Group Man Incobotulinum Toxin A Dosage (SD)	Group Mean Abobotulinum Toxin A Dosage (SD)
Minimum	81.62 (42.42)	85.94 (39.76)	237.50 (90.77)
Median	169.12 (73.70)	178.13 (65.75)	470.83 (171.17)
Maximum	311.76 (125.66)	328.13 (109.50)	779.17 (279.17)
Respondents	17	16	12

## Data Availability

The data presented in this study are available upon request from the corresponding author. It is typical for a survey to share on request, as it is hard to interpret.
